# An Autocrine IL-6/IGF-1R Loop Mediates EMT and Promotes Tumor Growth in Non-small Cell Lung Cancer

**DOI:** 10.7150/ijbs.31999

**Published:** 2019-07-20

**Authors:** Xianan Zheng, Guohua Lu, Yinan Yao, Wei Gu

**Affiliations:** 1 Department of Endocrinology, the Second Affiliated Hospital of Zhejiang University School of Medicine, Hangzhou, China; 2 Department of Respiratory Diseases, the First Affiliated Hospital of Zhejiang University School of Medicine, Hangzhou, China

**Keywords:** IGF-1, STAT3, epithelial-to-mesenchymal, transition, IL-6, NSCLC

## Abstract

Epithelial-to-mesenchymal transition (EMT) is a key process in EGFR-TKI resistance but the detailed mechanism is largely unknown. We aim to evaluate the role of interleukin-6 (IL-6) and insulin-like growth factor-1 receptor (IGR-1R) in EMT in non-small cell lung cancer (NSCLC). We used IL-6 to induce EMT in EGFR-TKI sensitive NSCLC cells. We found that both STAT3 and IGF-1R were activated. Interestingly activation of STAT3 and JAK1 was blocked by inhibiting IGF-1R, suggesting that IGF-1R might signal via JAK/STAT3. Activation of IGF-1R and AKT was inhibited by blocking STAT3, suggesting that STAT3 blockade might provide negative feedback signal to inhibiting IGF-1R. Reporter assay further confirmed that STAT3 activated gene transcription of IGF-1R. RT-PCR analyses showed that IL-6 induced the expression of IL-6 per se as well as IGF-1 and IGF-2. Expression of IL-6 and IGF-1R ligands was suppressed by inhibiting either STAT3 or IGF-1R. Meanwhile IL-6 induced gefitinib resistance and increased migration. We elucidated an autocrine loop of IL-6/IGF-1R/STAT3 in EMT-mediated resistance and tumor growth in NSCLC.

## Introduction

Non-small cell lung cancers (NSCLCs) that harbour activating mutations such as exon 19 deletion mutation are particularly sensitive to EGFR-TKIs gefitinib (Iressa) and erlotinib (Tarceva). Unfortunately, all NSCLC patients with erlotinib/gefitinib-sensitizing mutations eventually acquire resistance after a median of 6-11 months of EGFR TKI therapy 1, 2. Epithelial-to-mesenchymal transition (EMT), as manifested by loss of E-cadherin and increased expression of vimentin, snail and β-catenin, was reported as an EGFR resistance mechanism in lung adenocarcinoma cell lines 3. Development of EMT was observed in a NSCLC patient, who acquired resistance to erlotinib in the absence of known resistance mechanisms, such as the EGFR T790M mutation and MET amplification 4.

Signaling through IGF-1R has an essential role in cell mitosis, survival, and transformation and has been associated with higher risk of multiple neoplasms 5. IGF-1 stimulates IGF-1R and the IGF-1R/insulin receptor (IGF-1R/IR) heterodimers. Recently the induction of an EMT phenomenon was found to proceed in part through the activation of IGF-1R, leading to desensitization of del E746-A750-mutated PC-9 cells to erlotinib 6. But the molecular mechanism underlying this IGF-1R mediated mesenchymal transformation is unknown.

IL-6 is a pleiotropic cytokine that has been implicated in proliferation and survival of tumor cells. A recent study found that IL-6 is sufficient to induce gefitinib resistance, acquisition of mesenchymal-like features and invasion ability through activation of STAT3 and AKT 7. IL-6/STAT3 axis activation has been associated with EMT and resistance to TKI in various studies 7, 8. We hypothesized that IL-6/STAT3 signaling pathway and IGF-1 axis might collaborate in the induction of EMT, and further resulting in TKIs resistance.

In the present study we demonstrated that IL-6 induced EMT of PC-9 cells through activation of STAT3 and IGF-1R. Interestingly, we found that IL-6 exposure stimulated the autocrine secretion of IL-6 as well as IGF-1 and IGF-2. STAT3 was critical in establishing the autocrine network of IL-6 and IGF axis. This study linked the pro-inflammatory cytokine IL-6 with endocrine IGF axis in facilitating EMT in lung cancer cells.

## Materials and Methods

### Cell lines and cell cultures

PC-9 cells were kindly provided by Prof. Caicun Zhou (Shanghai Pulmonary Hospital, Shanghai, China). For all experiments, cells were cultured in RPMI 1640 medium supplemented with 10% fetal bovine serumand incubated in a humidified incubator at 37°C and 5% CO2. Cells were treated with recombinant human IL-6 50ng/mL (PeproTech, Rocky Hill, USA) for 48 hours. The inhibitors, dissolved in dimethyl sulfoxide (DMSO), were used in the following concentrations: STAT3 inhibitor AG-490 (50uM/L) (Selleck Chemicals, Houston, USA), IGF-1R inhibitor AG-1024 (5uM/L) (Selleck Chemicals), AKT inhibitor MK-2206 2HCl (5uM/L) (Selleck Chemicals, Houston, USA), JAK1 inhibitor ZM 39923 HCl (50uM /L) (Selleck Chemicals, Houston, USA). Human IGF-I antibody (R&D Systems) was used at the concentration of 10ug/ml. Subcultures were produced by trypsinization and were reseeded for experiments. Protein lysates were subjected to western blot analysis.

### Migration assay

Transwell insert chambers with an 8-μm porous membrane (Corning Costar, Cambridge, MA, USA) were used for the assay. Cells were washed three times with PBS and 1x10^5^ PC-9 cells were added to the top chamber in serum-free media. The bottom chamber was filled with media containing 50ng/ml IL-6. PC-9 Cells and A549 cells were incubated for 18 h and 24 h respectively at 37˚C in a 5% CO2 humidified incubator. To quantify the number of migratory cells, cells on the top chamber were removed with a cotton-tipped swab, and migrated cells were fixed in methanol and stained with 1% crystal violet. Five random fields were counted.

### Flow cytometric analysis

Cells were trypsinized, suspended into single-cell mixtures, washed with phosphate buffered saline (PBS), and incubated on ice for 30 min with monoclonal antibodies specific for human cell surface markers Annexin-FITC (eBioscience, San Diego, CA, USA) or PI (eBioscience). Cells were washed and analyzed using a flow cytometer (BD FACS Aria, San Jose, CA, USA).

### Western blot

For western blot analysis, differentially treated cells were washed with cold PBS and then lysed in lysis buffer containing protease and phosphatase inhibitor. Insoluble cell fragments were removed by centrifugation. Cells were separated by SDS-PAGE electrophoresis and next transferred to PVDF membranes. Membranes were incubated overnight with the primary antibodies against phospho-AKT (Cell Signaling Technologies), ERK-1/2 (Cell Signaling Technologies), phosphor-ERK-1/2 (Cell Signaling Technologies), STAT3 (Cell Signaling Technologies), phospho-STAT3 (Cell Signaling Technologies), phospho-IGF-I (Cell Signaling Technologies, Danvers, MA, USA), JAK1, phospho-Jak1 (Tyr1022/1023) (Cell Signaling Technologies), JAK2 (Cell Signaling Technologies), phospho-Jak2 (Tyr1007/1008) (Cell Signaling Technologies), IGF-I Receptor β (Cell Signaling Technologies), AKT (Cell Signaling Technologies), epithelial-mesenchymal transition (EMT) antibody sampler kit (Cell Signaling Technologies). Fluorescent secondary anti-mouse (Thermo Scientific, Waltham, USA) and anti-rabbit (Thermo Scientific, Waltham, USA) antibodies were used and were detected using Odyssey Sa infrared imager (LI-COR Biosciences, Lincoln, USA).

### Real-time RT-PCR

Differentially treated cells were subjected to RNA extraction using Total RNA Isolation Reagent (Takara Bio, Japan) per manufacturer's instructions. Synthesis of cDNA with reverse transcriptase was performed by PrimeScript™ RT reagent Kit (Perfect Real Time) (Takara Bio, Japan). RT-PCR was performed using the SYBR® Premix Ex Taq™ II (Tli RNaseH Plus) (Takara Bio, Japan) on CFX96 Real-Time PCR Detection System (Bio-Rad, USA). Primers used for RT-PCR were as follows: 5'-ACTCACCTCTTCAGAACGAATTG-3' (forward) and 5'-CCATCTTTGGAAGGTTCAGGTTG-3' (reverse) for IL-6; 5'-TCGACATCCGCAACGACTATC-3' (forward) and 5'-CCAGGGCGTAGTTGTAGAAGAG-3' (reverse) for IGF-1; 5'-GTGACCAGCAAGGCACAAATC-3' (forward) and 5'-CACCAAGTAGGCACCACTAAG-3' (reverse) for IGF-2; and 5'-TCATGAAGTGTGACGTTGACATCCGT-3' (forward) and 5'-CCTAGAAGCATTTGCGGTGCACGATG-3' (reverse) for β-actin. The PCR program was 95˚C for 30 sec, followed by 40 cycles of 95˚C for 5 sec and 60˚C for 30 sec. A standard melting-curve analysis was carried out at the end of the amplification. Each RT-PCR experiment was done in triplicates. All the mRNA expression values were normalized to an internal control β-actin.

### Luciferase reporter assay

Luciferase reporter gene assays were performed using the Dual-Luciferase Reporter Assay System (Hanbio Biotechnology, China), according to the manufacturer's instructions. The 293 T cells were transfected for 48 h, with a pGL3-basic, STAT3, PCDNA3.1 and IGF1R-pro vector, respectively. Thereafter, firefly and Renilla luciferase activities were measured continuously using a dual luciferase reporter assay system (Tecan Infinite M1000 PRO, Switzerland). Finally, firefly to Renilla luciferase ratios were calculated for each well, and each measurement was repeated three times in three independent experiments.

### ELISA

PC-9 cells were cultured in 6-well plates and after 48 hours, culture supernatants were harvested. IGF-1 levels in culture supernatants were measured using the IGF-1 Quantikine human ELISA kits (R&D Systems) in accordance with the manufacturer's instructions.

### Statistical Analysis

All reported values were presented as the mean values with 95% confidence intervals. At least three independent experiments were done to obtain each result and statistical comparisons among groups were determined using one-way ANOVA. Two-sided P values of<0.05 were considered statistically significant.

## Results

### IL-6 induces EMT in time and concentration dependent manner

**Figure 1:**
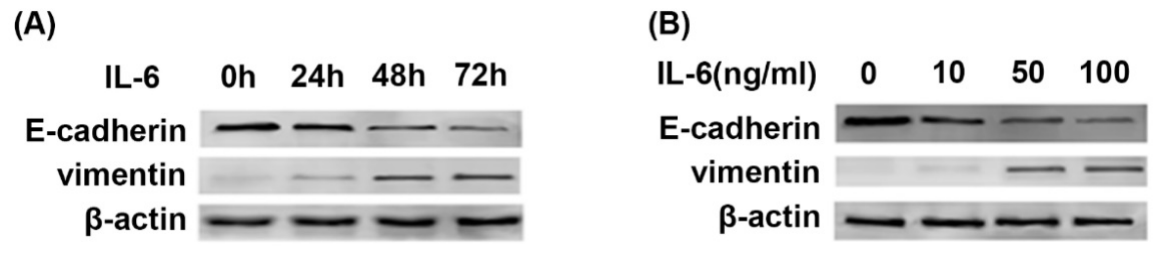
** IL-6 induces EMT in time and concentration dependent manner. (A)** PC-9 cells were treated with IL-6 (50ng/ml) for an indicated period of time and harvested for western blot of E-cadherin and vimentin. **(B)** Cells were treated with the indicated concentration of IL-6 and harvested after 48 hours for western blot.

Previous studies showed that the stimulation of TKI-sensitive cells by IL-6 decreased erlotinib sensitivity 8. Since EMT is one of major mechanisms of acquired resistance, we were motivated to investigate whether IL-6 could induce EMT in TKI-sensitive PC-9 cells. Western blot showed that IL-6 promoted EMT, as presented by repression of E-cadherin and concomitant induction of Vimentin compared with control cells in time and concentration-dependent manner (Fig. 1A and 1B). The most significant change of EMT markers was seen at 48 and 72 hours (Fig. 1A) and at a concentration of 50ng/ml and 100ng/ml (Fig. 1B). Thus, for the following experiments we used IL-6 at 50ng/ml and analyzed molecular changes after 48 hours of incubation.

### IL-6 induces EMT through activation of STAT3

**Figure 2:**
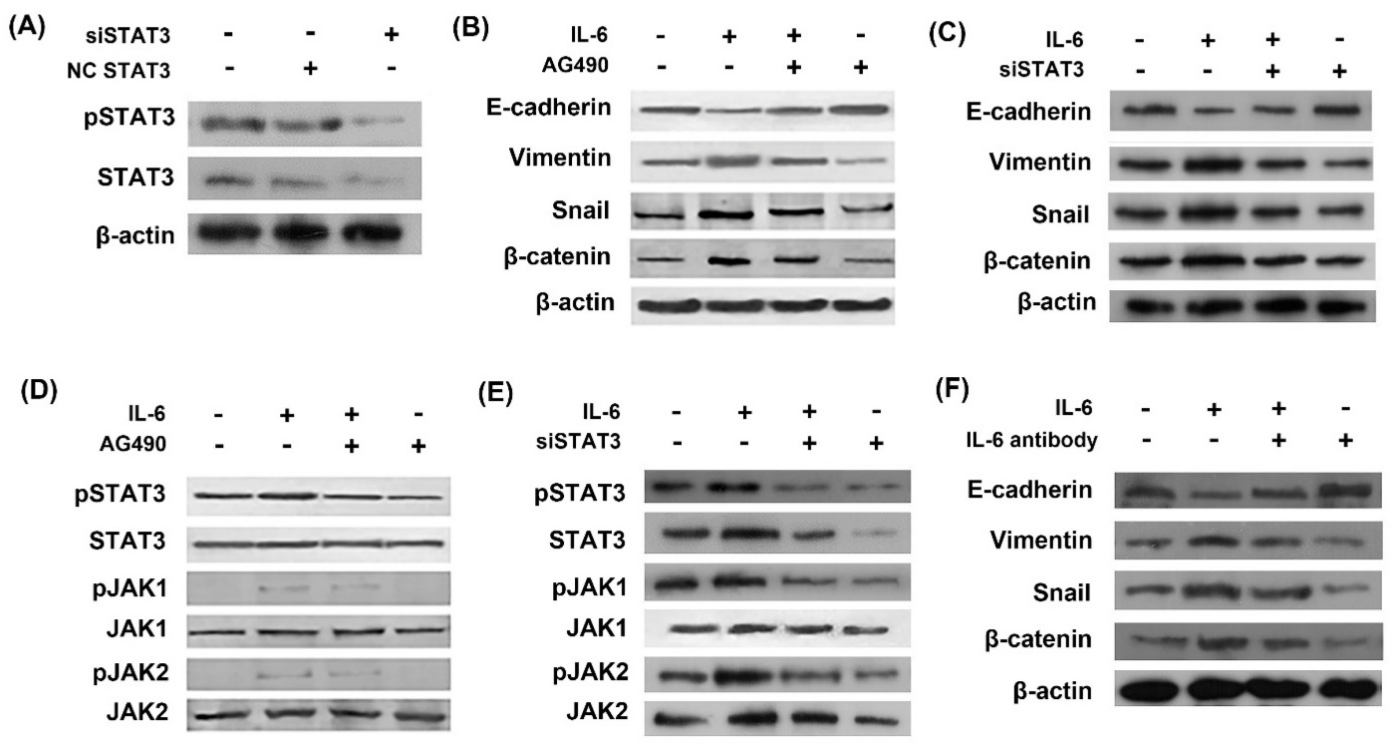
** IL-6 induced EMT through activation of STAT3.** Cells were transfected with either STAT3 siRNA (siSTAT3) or negative control siRNA (NC siRNA) and activation of STAT3 was measured by western blot **(A)**. Cells were incubated with IL-6 (50ng/ml) with or without AG 490 (50uM/L) for 48 hours and harvested for western blot of EMT markers **(B)** and JAK/STAT3 signaling molecules **(D)**. Cells were transfected with siSTAT3 and EMT markers **(C)** and JAK/STAT3 signaling molecules **(E)** were measured by western blotting. Cells were treated with IL-6 with or without IL-6 antibody and EMT markers were measured **(F)**. Blots were representatives of three experiments.

In order to study the role of STAT3 in EMT, we used STAT3 siRNA (siSTAT3) to silence STAT3 gene. The inhibitory effect of siSTAT3 was confirmed by western blots as STAT3 was inhibited using siSTAT3 compared with negative control siRNA (Fig. 2A). Western blot showed that IL-6 induced EMT, as evidenced by induction of mesenchymal markers Vimentin, snail and β-catenin and repression of the epithelial marker E-cadherin (Fig. 2B and 2C). STAT3 was activated by IL-6 exposure in the induction of EMT and inhibited by AG490 or siSTAT3 (Fig. 2D and 2E). Blocking STAT3 with AG490 or siSTAT3 eliminated the effect of IL-6 to induce EMT, as E-cadherin increased and Vimentin and β-catenin decreased compared to the cells treated with IL-6 alone (Fig. 2B and 2C).

We next investigated whether neutralizing antibody of IL-6 reversed EMT. As a result, IL-6 antibody blocked EMT, as shown by increased E-cadherin and decreased Vimentin, snail and β-catenin compared to cells treated with IL-6 alone (Fig. 2F).

### IL-6 induces EMT through cross-activation of IGF-1R

**Figure 3:**
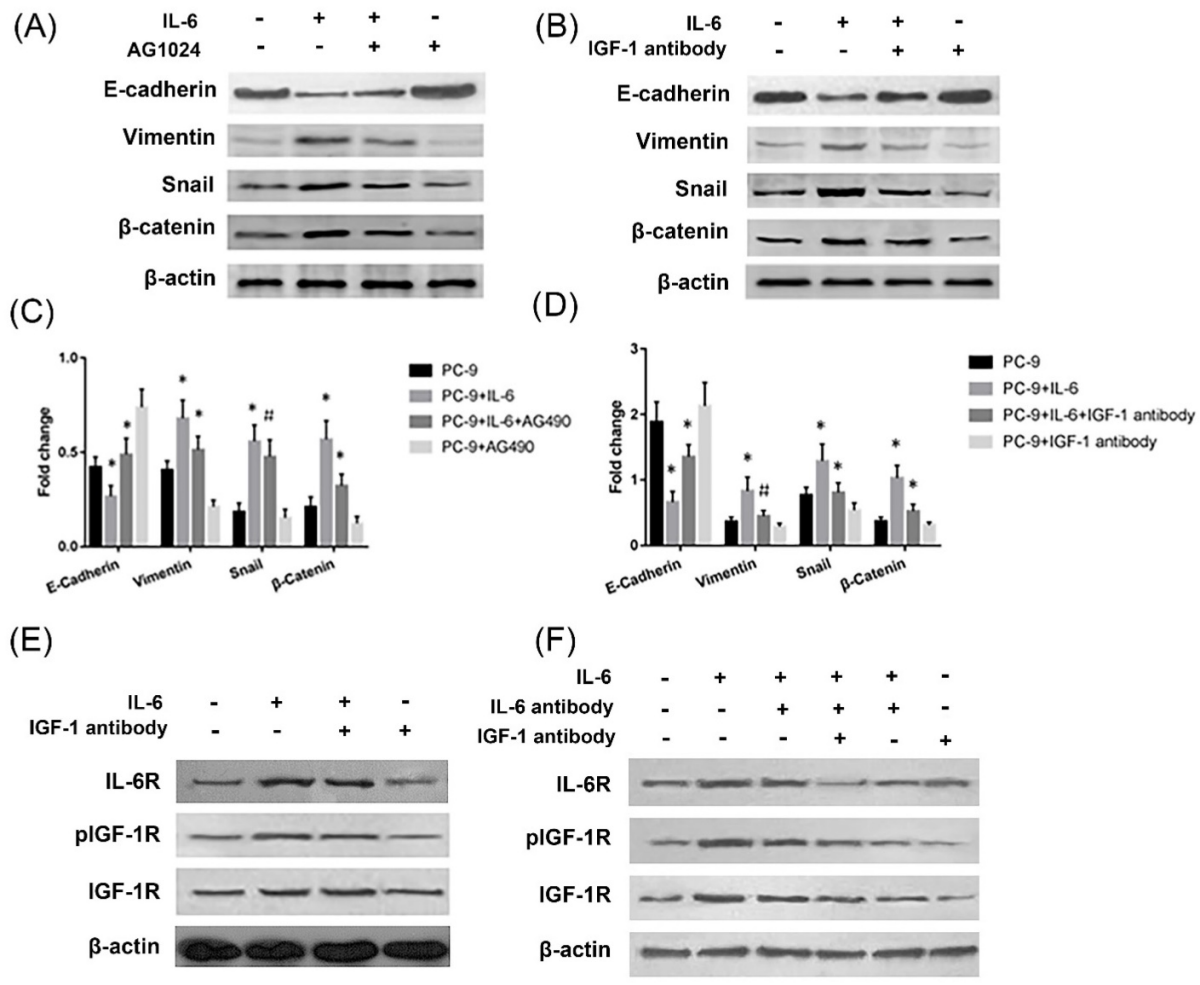
** IL-6 induced EMT through activation of IGF-1R.** Cells were treated with IL-6 (50ng/ml) in the presence of IGF-1R inhibitor AG1024 (5uM/L) **(A and C)** or IGF-1 neutralizing antibody (10ug/ml)** (B and D)** and harvested after 48 hours for western blotting of EMT markers. Activation of IL-6R and IGF-1R was measured in the presence of IGF-1 antibody **(E)** or when the neutralizing antibody of both IGF-1 and IL-6 were present **(F)**. Blots were representatives of three experiments and results were quantified using Quantity One for comparisons between IL-6 group and control or between IL-6 plus an inhibitor (AG1024 or IGF-1 antibody) co-treated cells and IL-6 group. *, p<0.05. #, p>0.05 not significant.

The effect of IL-6 to induce EMT was blocked by IGF-1R inhibitor AG1024, as evidenced by increased E-cadherin and decreased Vimentin and snail levels compared to cells treated with IL-6 alone (Fig. 3A and 3C). Β-catenin did not show a significant change upon AG1024 treatment. We further investigated whether neutralizing antibody of IGF-1 affected EMT. As a result, IGF-1 antibody blocked EMT, as shown by increased E-cadherin and decreased Vimentin, snail and β-catenin levels compared to cells treated with IL-6 alone (Fig. 3B and 3D). IL-6 might induce EMT through activation of IGF-1R.

On the other hand, IL-6 activated IGF-1R and IL-6R. Neutralizing antibody of IGF-1 or IL-6 alone has no significant impact on IL-6R or IGF-1R activation, while dual inhibition of IL-6 and IGF-1 with neutralizing antibodies significantly inhibited IL-6R (Fig. 3E and 3F). We concluded that endogenous IGF-1 and IL-6 secreted from PC-9 cells might promote EMT through activation of IGF-1R and IL-6R/STAT3.

### IL-6 and IGF-1R cross signal to promote EMT

**Figure 4:**
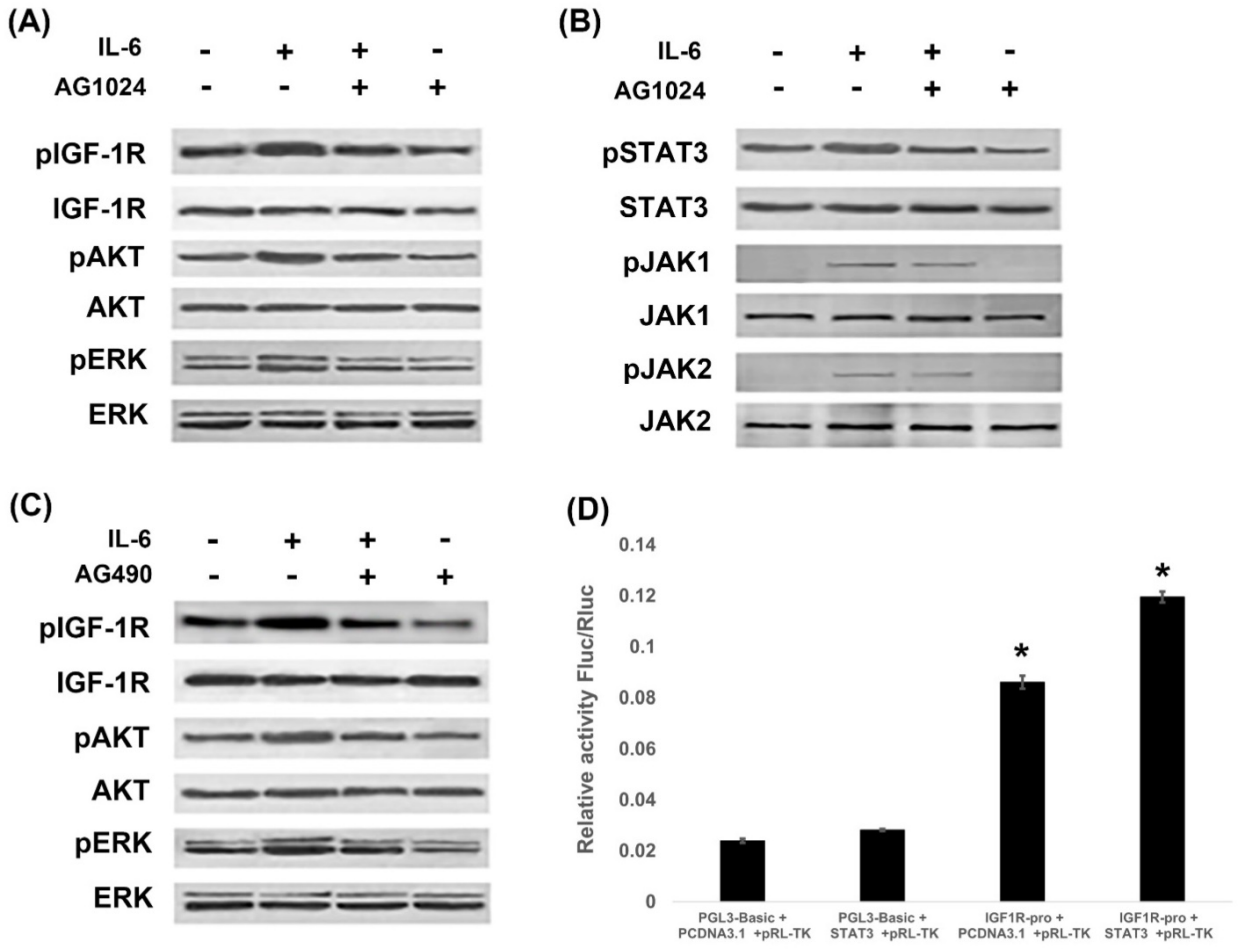
** IL-6 and IGF-1R cross-signal to promote EMT.** PC-9 cells were treated with IL-6 with or without IGF-1R inhibitor AG1024 for 48 hours, and IGF-1R pathway **(A)** and JAK/STAT3 pathway** (B)** were tested using western blot. Cells were treated with IL-6 with or without STAT3 inhibitor AG490 for 48 hours, and IGF-1R pathway were tested using western blot **(C)**. STAT3 over-expressed plasmids were constructed and promoter activity of IGF-1R was studied by reporter assay** (D)**. Blots were representatives of three experiments and results were quantified using Quantity One for comparisons between IL-6 group and control or between IL-6 plus an inhibitor co-treated cells and IL-6 group. *, p<0.05. #, p>0.05 not significant.

Since IL-6 induced EMT through activation of STAT3 and IGF-1R, we next investigated whether IL-6/STAT3 and IGF-1R were cross-linked to promote EMT. PI3K/AKT, ras-raf-MEK-ERK and JAK/STAT3 are signal transduction pathways common to IL-6 and IGF-1. We found that IL-6 activated IGF-1R, AKT and ERK, while blocking IGF-1R with AG1024 inhibited IGF-1R and ERK1/2 (Fig. 4A). On the other hand, IL-6 activated STAT3 along with JAK1 and JAK2 (Fig. 4B), while blocking STAT3 by AG490 inhibited STAT3, but not JAK1 or JAK2 (Fig. 2D). Interestingly, blocking IGF-1R inhibited STAT3 and JAK1 (Fig. 4B), while blocking STAT3 inhibited IGF-1R and AKT (Fig. 4C).

We further examined the contributions of IGF-1R and IL-6/STAT3 pathways using reporter assay. STAT3 over-expressed plasmids were constructed and the gene transcription of IGF-1R was studied. It was shown that STAT3 increased the promoter activity of IGF-1R (Fig. 4D). We confirmed that STAT3 impacted IGF-1 signaling on a genetic level.

### IL-6/IGF-1R autocrine loop is mediated via STAT3

**Figure 5:**
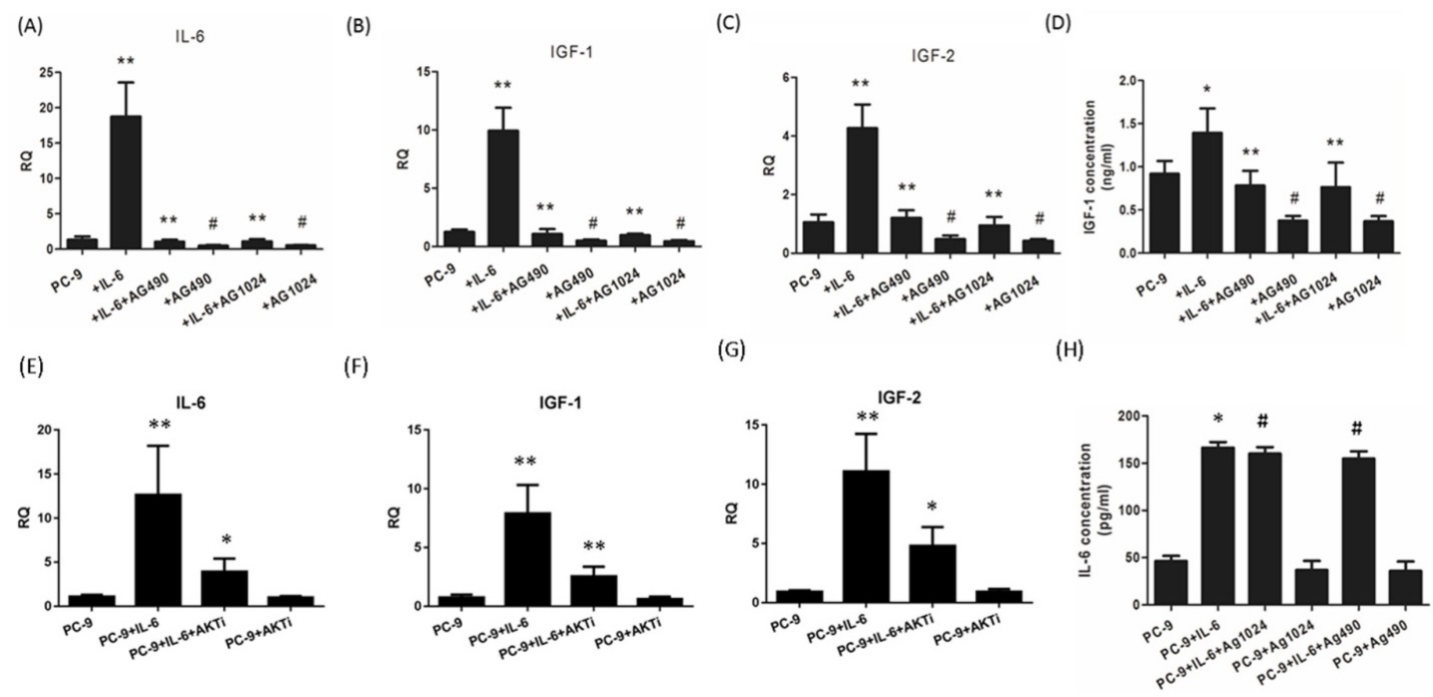
** IL-6/ IGF-1R autocrine loop is mediated via STAT3.** Cells were treated with IL-6 with or without an inhibitor (AG490, AG1024 or AKT inhibitor MK-2206 2HCl) and RT-PCR analyses were performed after 48 hours. Gene expressions of IL-6 **(A and E)**, IGF-1 **(B and F)** and IGF-2 **(C and G)** were compared between IL-6-treated cells and control group and between IL-6 plus inhibitor co-treated cells and IL-6 group. ELISAs were performed for IGF-1 **(D)** and IL-6 **(H)** and results were compared in the same way as the above mentioned RT-PCR analyses. *, p<0.05. **, p<0.01. #, p>0.05 not significant.

Since endogenous IGF-1 and IGF-2 take effect through IGF-1R signaling, we were motivated to explore whether IL-6 could induce the secretion of IGF-1R ligands during EMT. Exogenous IL-6 stimulated the mRNA expression of IL-6 as well as IGF-1 and IGF-2 (Fig. 5A to C). In the presence of STAT3 or IGF-1R inhibitor, IL-6 showed little effect on IL-6 expression and IGF axis, as evidenced by decreased expression of IL-6, IGF-1 and IGF-2 compared to IL-6-treated cells. ELISA confirmed that IL-6 stimulated the secretion of IGF-1 and IL-6. Blocking either STAT3 or IGF-1R reduced the secretion of IGF-1 but not IL-6 (Fig. 5D and 5H).This was probably because IL-6 increased the secretion of IL-6 at a level of approximately 100pg/ml, which was too low compared to the exogenous IL-6 (50ng/ml) to elicit a statistically significant result. These data indicated that IL-6 might stimulate the autocrine secretion of IL-6 and IGF-1R ligands, cross-activate IGF-1R and ultimately STAT3 leading to EMT.

In the present study, we found that AKT was slightly activated by IL-6 and inhibited by blocking either IGF-1R or STAT3. Since AKT was regarded as one of two major pathways of IGF-1R signaling, we suspected that AKT might coordinate the cross-talk between IL-6 and IGF-1R. We found that blocking AKT significantly reduced the expression of IL-6, IGF-1 or IGF-2 (Fig. 5E, F and G). The autocrine loop of IL-6/IGF-1R might be mediated by AKT apart from STAT3.

### IL-6 induced gefitinib resistance and increased migration via STAT3 and IGF-1R

**Figure 6:**
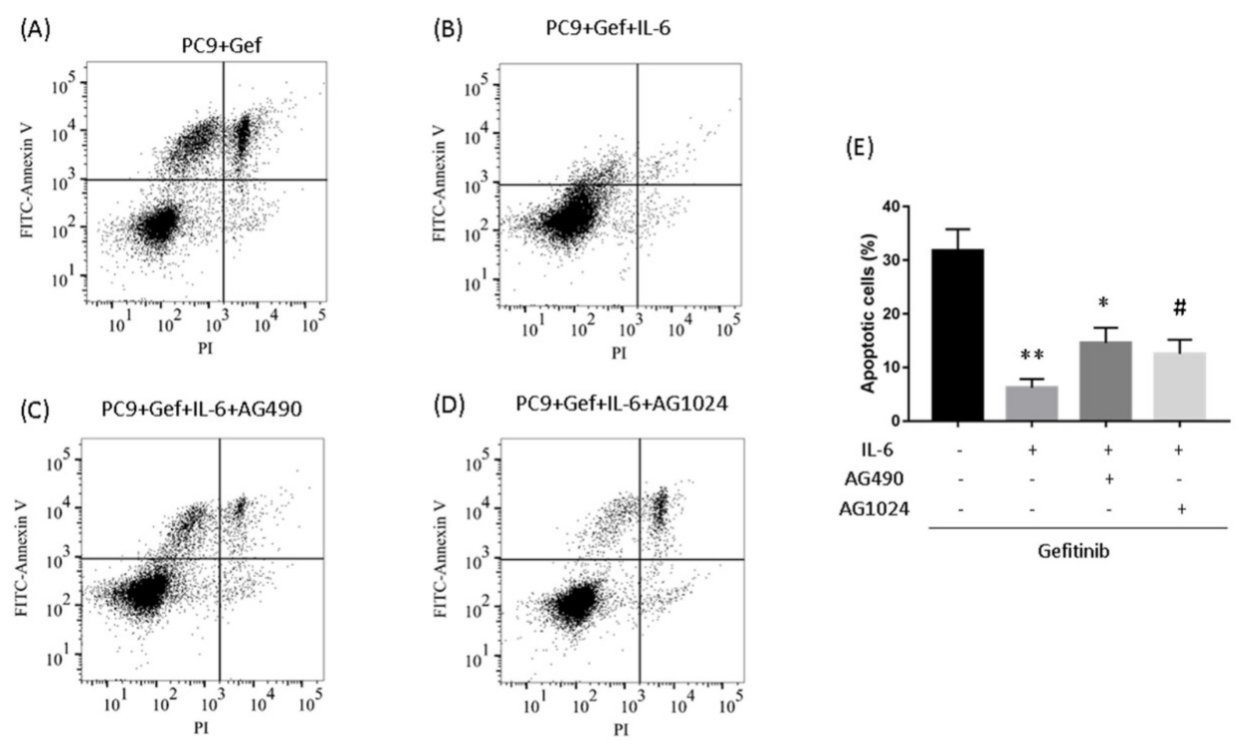
**Inhibiting STAT3 partly restores gefitinib sensitivity. (A to D)** PC-9 cells were treated with gefitinib (50nmol/L) for 48 h. Cells were stained with annexin V-FITC and PI and subsequently analyzed using flow cytometry. **(E)** Quantification of gefitinib-induced apoptotic cells by annexin/PI double-staining. Apoptosis was evaluated by determining the percentage of annexin V-positive cells. The data are representative of three independent experiments and compared between IL-6 treated cells and gefitinib group, or between IL-6 plus an inhibitor co-treated cells and IL-6 group. *, p<0.05. **, p<0.01. #, p>0.05 not significant.

**Figure 7:**
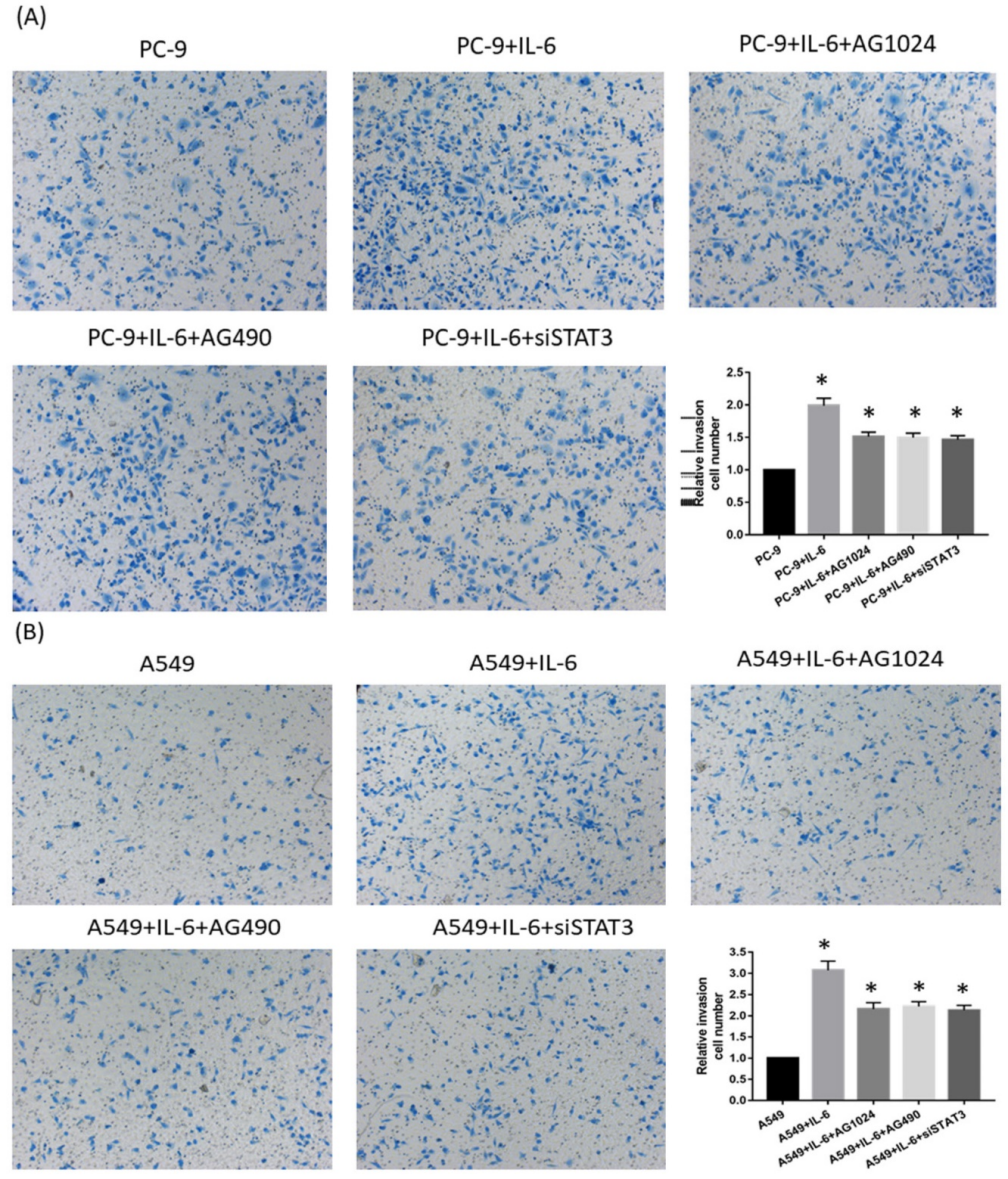
** Inhibiting either STAT3 or IGF-1R attenuates cell migration.** PC-9 cells **(A)** and A549 cells **(B)** were seeded and treated either with IL-6 alone or in the presence of both IL-6 and an STAT3 or IGF-1R inhibitor. Migration was shown as photograph and the number of migratory cells was determined using a transwell matrix penetration assay and quantified for analysis of significance. *, p<0.05. #, p>0.05 not significant.

To determine whether IL-6-induced gefitinib resistance, we analyzed proportion of apoptosis using flow cytometry. Following 48 hours of IL-6 exposure, gefitinib-induced apoptotic cells significantly reduced, suggesting IL-6 decreased gefitinib sensitivity (Fig. 6A and 6B). EGFR-TKI sensitivity was partly restored by blocking STAT3 (Fig. 6C and E). Inhibiting IGF-1R increased the apoptotic cell count but the difference was not significant (Fig. 6D and E). Meanwhile, IL-6 increased the migration of PC-9 (Fig. 7A) and A549 cells (Fig. 7B). Blocking either STAT3 or IGF-1R attenuated the effect of IL-6 to induce migration in PC-9 (Fig. 7A) and A549 cells (Fig. 7B). These data indicated that IL-6 induced gefitinib resistance and migratory ability through activation of STAT3 and IGF-1R.

## Discussion

EMT is a key process in embryonic development and has been associated with acquired resistance to the EGFR tyrosine kinase inhibitors 4. In the present study, we used interleukin-6 to induce EMT-associated gefitinib resistance and cell migration. We found that during the acquisition of EMT, both IGF-1R and STAT3 were activated. Inhibition of either IGF-1R or STAT3 abolished the effect of IL-6 to induce EMT, as well as the associated gain of drug resistance and migratory ability.

IGF-1 has been linked to EGFR TKI resistance and EMT in various preclinical studies. Sharma SV and colleagues found that chromatin modification mediated by IGF-1 signaling conferred resistance to erlotinib 10. A recent research discovered hyper-activation of IGF-1R and an EMT morphological change in erlotinib-resistant PC-9 cells 6. Inhibition of IGF-IR activation and knockdown of survivin expression led to increased apoptosis 11. STAT3 activation has been identified in patients resistant to EGFR-TKI treatment 12. In the present study, we demonstrated that EMT was mediated through activation of STAT3 apart from IGF-1R. STAT3 and IGF-1R might be linked to promote EMT.

Intriguingly, we observed decreased activation of IGF-1R as well as AKT and ERK by blocking STAT3, and decreased activation of STAT3 and JAK1 not JAK2 after blocking IGF-1R. We assumed that IGF-1R might signal via JAK/STAT3. Decreased phospho-STAT3 might provide negative feedback signal to inhibiting IGF-1R. STAT3 might be an important coordinator between IGF-1R and IL-6 signaling. Previous studies have found that IGF-1 stimulates STAT1 and STAT3 in neurons, cardiomyocytes and other cell types 13; 14; 15. To our knowledge, there are few studies demonstrating that IGF-1R signals through STAT3 in promoting EMT in lung cancer cells.

In the present study we found that IL-6 activated STAT3, as well as JAK1 and JAK2. Besides, JAK1 instead of JAK2 was blocked by inhibiting IGF-1R. On one hand, JAK2/STAT3 was found to mediate tumor angiogenesis in NSCLC 16 and EMT in ovarian carcinomas 17. On the other hand, JAK1 has been proved to associate with EGFR TKI resistance. Activation of IL-6R/JAK1/STAT3 was shown to confer afatinib resistance in NSCLC cells with EGFR T790M 18. Future researches are needed to determine which member of the JAK family is involved in IL-6 and IGF-1R cross-talk in NSCLC.

An interesting finding from our present study was that IL-6 increased the expression of IGF-1R ligands IGF-1 and IGF-2, as well as IL-6 itself. It seemed that IL-6 stimulated the autocrine secretion of IL-6, which formed a positive feedback loop to activate IL-6R and IGF-1R, further stimulated the secretion of IGF-1 and IGF-2. By binding to its ligands, IGF-1R was activated, leading to EMT through downstream effectors of STAT3 as well as AKT and ERK. This positive feedback loop was interrupted when either STAT3 or IGF-1R was inhibited, probably due to a positive role of STAT3 on IL-6/IGF-1R auto-secretion. STAT3 increased gene transcription of IGF-1R as proved by dual luciferase reporter assay.

The autocrine loop of IL-6/STAT3 has been proposed previously. In tumor cells, STAT3-mediated upregulation of sphingosine-1-phosphate receptor-1 led to persistent STAT3 activity, forming a positive feed-forward loop 19. STAT3 inhibition in cancer-derived cell lines resulted in a significant effect on in vivo tumor growth, possibly through a reduction in the STAT3-mediated production of tumor-secreted IL-6 20. However, there are few studies connecting IGF-1R to IL-6/STAT3 loop with an emphasis on its role in inducing EMT. A recent study on cardiomyocytes show that expression of IL-6 was increased in aged wild-type mice, while this increase was attenuated in aged IGF-1R knockout mice, suggesting an essential role of IGF-1R in regulating IL-6 expression and cardiomyocyte senescence 21.

Since PI3K-AKT is one of the two canonical IGF-1R signal transduction pathways, with the other one being the ras-raf-MEK-ERK pathway 22, we tested whether AKT was another coordinator of IGF-1R and IL-6 signaling. We observed that AKT was slightly activated in acquiring EMT and partially inhibited by blocking either IGF-1R or STAT3. Blocking AKT significantly reduced the expression of IL-6, IGF-1 and IGF-2. AKT is a proto-oncogene and its expression and activation has been shown to increase in NSCLC 23. Besides, AKT was also demonstrated to involve in the resistance of cancer cells to chemotherapy and radiation treatment 24. Whether AKT plays a part in mediating EMT and the IL-6/IGF-1R loop needs further confirmation.

In the present study we showed that IL-6 decreased gefitinib sensitivity, as gefitinib-induced apoptosis reduced upon IL-6 exposure. EGFR-TKI sensitivity was partly restored by blocking STAT3. A previous study reported that in both H1975 and PC9-GR cells, IL-6 mRNA levels and IL-6 secretion were elevated upon afatinib treatment, while blocking IL-6R significantly increased the anti-proliferative effect of afatinib. This indicated that afatinib resistance was mediated by activation of IL-6R signaling via autocrine IL-6 production 18. So we considered that IL-6 may elicit pro-tumor effect on both gefitinib-sensitive and gefitinib-resistant NSCLC cell lines.

In summary, we demonstrate the utility of IL-6-induced EMT models in vitro to identify potential therapeutic targets in this otherwise largely treatment-refractory subpopulation. We show that IGF-1R has an important role in inducing EMT, probably through activation of STAT3. An interestingly finding from our study is that there exists an autocrine loop of IL-6/IGF-1R, in which IL-6 may stimulate gene expression of IL-6 itself as well as IGF-1 and IGF-2. STAT3 might in turn facilitate this positive feedback loop. Our pre-clinical study may provide evidence in establishing combination therapy targeting both STAT3 and IGF-1R for drug resistant patients that undergo EMT.

## Abbreviations

IL-6: interleukin-6
IGF-1: insulin-like growth factor-1
EMT: Epithelial to mesenchymal transition
EGFR: epidermal growth factor receptor
NSCLC: non-small cell lung cancer
